# Sexual disorders and psychotropic drugs :about 250 cases

**DOI:** 10.1192/j.eurpsy.2023.239

**Published:** 2023-07-19

**Authors:** L. tbatou, B. yassmine, L. fouad

**Affiliations:** Psychiatric emergency, Hospital ArRazi, salé, Morocco

## Abstract

**Introduction:**

The effect of psychotropic drugs on sexuality is difficult to evaluate because psychiatric disorders are frequently accompanied by sexual dysfunction, independently of any drug intake. However, the iatrogenic aspects of psychotropic drugs linked to their side effects should not be underestimated since they are an important cause of treatment discontinuation.

**Objectives:**

The aim is to show the type of sexual impairment observed according to the different psychotropic drugs used and the means to remedy it. Psychiatric disorders are accompanied by sexual dysfunctions which can be aggravated by psychotropic drugs, and the associated risk factors can be sought or aggravate this undesirable effect.

**Methods:**

This is a cross-sectional study, concerning patients followed at ArRazi hospital under antipsychotic treatment. We used the “International Index of Erectile Dysfunction” scale, and the data were analyzed by SPSS software.

**Results:**

68% are men, Age: 71% are between 20 and 50 years old.

Marital status: 43% are single, 24% are married, 34% are divorced.

83% have a low socioeconomic level, 45% have a family history of a psychiatric disorder.

58% of patients have a substance use disorder.

38% of patients consulted for a psychotic syndrome, 23% for a depressive syndrome, 17% for a manic syndrome, 11% for a behavioral disorder, 6% for a suicide attempt.

46% of the medications are antipsychotics, 18% are antidepressants, 15% are mood regulators.

83% of the sexual disorder appeared in the first 5 years.

72% of the patients did not receive information by their doctor about this side effect.

64% of the patients informed their doctor.

32% stopped the treatment, 47% asked to change the treatment.

**Conclusions:**

Careful assessment of sexual function at the initial visit followed by monitoring at subsequent visits is essential. Treatment of adverse sexual effects may be pharmacological, behavioral, complementary and integrative, or a combination of these modalities

**Disclosure of Interest:**

None Declared

